# The role of oxidative stress in streptozotocin-induced diabetic nephropathy in rats

**DOI:** 10.1590/2359-3997000000188

**Published:** 2016-08-23

**Authors:** Sheila Marques Fernandes, Priscilla Mendes Cordeiro, Mirian Watanabe, Cassiane Dezoti da Fonseca, Maria de Fatima Fernandes Vattimo

**Affiliations:** 1 Laboratório Experimental de Modelos Animais Escola de Enfermagem Universidade de São Paulo São Paulo SP Brasil Laboratório Experimental de Modelos Animais (LEMA), Escola de Enfermagem da Universidade de São Paulo (EEUSP), São Paulo, SP, Brasil; 2 Universidade Federal do Amazonas Manaus AM Brasil Colegiado de Enfermagem, Universidade Federal do Amazonas (UFAM), Manaus, AM, Brasil

**Keywords:** Oxidative stress, rats, diabetic nephropathy, uninephrectomy

## Abstract

**Objective:**

The objective of this study was to evaluate the role of oxidative stress in an experimental model of streptozotocin-induced diabetic nephropathy in rats.

**Materials and methods:**

Wistar, adult, male rats were used in the study. Animals were divided in the following groups: Citrate (control, citrate buffer 0.01M, pH 4.2 was administrated intravenously - i.v - in the caudal vein), Uninephrectomy+Citrate (left uninephrectomy-20 days before the study), DM (streptozotocin, 65 mg/kg, i.v, on the 20^th^ day of the study), Uninephrectomy+DM. Physiological parameters (water and food intake, body weight, blood glucose, kidney weight, and relative kidney weight); renal function (creatinine clearance), urine albumin (immunodiffusion method); oxidative metabolites (urinary peroxides, thiobarbituric acid reactive substances, and thiols in renal tissue), and kidney histology were evaluated.

**Results:**

Polyphagia, polydipsia, hyperglycemia, and reduced body weight were observed in diabetic rats. Renal function was reduced in diabetic groups (creatinine clearance*,* p < 0.05). Uninephrectomy potentiated urine albumin and increased kidney weight and relative kidney weight in diabetic animals (p < 0.05). Urinary peroxides and thiobarbituric acid reactive substances were increased, and the reduction in thiol levels demonstrated endogenous substrate consumption in diabetic groups (p < 0.05). The histological analysis revealed moderate lesions of diabetic nephropathy.

**Conclusion:**

This study confirms lipid peroxidation and intense consumption of the antioxidant defense system in diabetic rats. The association of hyperglycemia and uninephrectomy resulted in additional renal injury, demonstrating that the model is adequate for the study of diabetic nephropathy.

## INTRODUCTION

An estimation by the World Health Organization considers that by 2030, approximately 366 million people will be diagnosed as carriers of diabetes mellitus (DM) all over the world, with an increase in mortality and morbidity rates due to complications of the disease (1). Microvascular changes are the main consequence of chronic hyperglycemia and lead to an imbalance in cell metabolism, with progressive lesions in several organs, such as kidneys, eyes, nerves, liver, and the vascular, immunological and gastrointestinal systems (2,3).

Diabetic nephropathy is a progressive disease that involves several mechanisms, with changes in glomerular hemodynamics, causing renal lesions, oxidative stress, inflammatory response, and fibrosis. Chronic hyperglycemia lead to glomerular hyperfiltration by means of vasodilation of the afferent arteriole in relation to the efferent arteriole, leading to increased hydrostatic pressure and greater passage of fluids through the glomerulus (2,3). Glomerular dysfunction is observed as microalbuminuria caused by changes in renal structure, such as thickening of the basal membrane, podocyte lesions, expansion of the mesangial matrix, which evolve to glomerular sclerosis and tubulointerstitial fibrosis associated with reduced glomerular filtration rates (GFR) (3,4).

The cumulative results of these transformations are caused by excess production of reactive oxygen species (ROS) mediated by chronic hyperglycemia. ROS generation in diabetic kidneys is caused by enzymatic and non-enzymatic systems that include glucose auto-oxidation, Fenton reaction catalyzed by unbound iron, and consumption of the endogenous antioxidant reserve. ROS include free radicals, such as superoxide anion (O2^∙-^) and hydroxyl radical (OH^∙-^), and non-radicals such as hydrogen peroxide (H_2_O_2_). Production of reactive nitrogen species, such as nitric oxide radicals (NO) is also important (5).

Development of complications related with diabetes takes place with ROS production, mainly O2^.-^, which induces cell dysfunction and oxidative lesion by means of protein denaturation, lipid peroxidation, and damage to mitochondrial DNA (5,6). These changes in renal cells, including glomerular endothelial cells, mesangial cells, and renal epithelial cells, lead to changes in ATP synthesis, intracellular calcium imbalance, and changes in cell membrane permeability that contribute to cell death by apoptosis or necrosis (5,7).

Recent studies demonstrated that quantification of new plasmatic and urinary markers may contribute to early diagnosis of the development of diabetic nephropathy. Among these markers, the most important ones nowadays are: transforming growth factor β, vascular endothelial growth factor, and protein kinase C, as well as ROS (4,7).

Given the still uncertain scenario showing the participation of redox mechanisms as determinant in chronic hyperglycemia lesions, knowledge on mediators, and physiopathological processes responsive to therapeutic maneuvers, such as antioxidant supplementation associated with intense glycemic control, will contribute considerably with reducing microvascular complications in diabetic patients. In general, these progresses will provide scientific and clinical bases to reduce morbidity and mortality in this population. Therefore, the objective of this study was to demonstrate the role of oxidative stress in an experimental model of streptozotocin-induced diabetic nephropathy in rats.

## MATERIALS AND METHODS

### Protocol

This is a descriptive, experimental, quantitative study *in vivo* carried out in the Laboratório Experimental de Modelo Animais da Escola de Enfermagem at Universidade de São Paulo (LEMA – EEUSP).

All procedures carried out in this study complied with the Ethical Principles for the Use of Experimental Animals determined by the Colégio Brasileiro de Experimentação Animal – COBEA – and were approved by the Ethics Committee on Animal Experimentation of the university – CEEA (060/2012).

Twenty-four Wistar, adult, male rats were used. They weighted from 250 to 300 grams and were divided into the following groups: *Citrate (Control)*: citrate buffer 0.01M pH 4.2, by intravenous route in the caudal vein (i.v.); *Uninephrectomy + Citrate (Nx+Citrato)*: left uninephrectomy before the study (20 days) and administration of citrate buffer; *DM*: DM induction by means of the administration of streptozotocin (STZ) diluted in citrate buffer 0.01M, pH 4.2, 65 mg/kg, i.v.; *Uninephrectomy + DM (Nx+DM)*: animals subjected to Nx and STZ administration.

After 48 hours of DM induction, blood glucose was determined using the Advantage kit (Advantage – Roche^®^, Brazil). Animals with blood glucose above 250 mg/dL were selected for the diabetic group. After that, body weight and blood glucose were assessed every week for 12 weeks (85 days). Animals were treated with 0.5-2 U of insulin NPH (Lilly, Brazil), daily, to maintain blood glucose between 300-500 mg/dL.

### Collection of biological samples

At the end of the protocol (85 days), animals were placed in individual metabolic cages for 24-hour urine collection to evaluate renal function and oxidative metabolites. After that, animals were anesthetized by intraperitoneal (i.p.) route with Thiopentax^®^ (Cristália, Brazil) (sodium thiopental: 40-50 mg/kg) for blood collection by means of a puncture in the abdominal aorta and later evaluation of renal function.

The right kidney was removed and prepared for histological sections stained with hematoxylin-eosin. Another aliquot was immediately cooled and stored at -80ºC for later quantification of antioxidant enzymes. At the end of the study, animals were euthanized using the ethical guidelines for handling of experimental animals mentioned above.

### Renal function/albuminuria/oxidative metabolites and renal histology

Renal function was evaluated by means of creatinine clearance. The colorimetric method by Jaffé was used to determine serum and urinary creatinine results. Creatinine clearance was calculated using the formula: creatinine clearance = urinary creatinine x urine flow in 24 hours/serum creatinine (8,9).

Albuminuria was assessed by means of radial immunodiffusion based on antibody reaction and precipitation using rabbit anti-rat albumin against albumin in the urine samples. Two standard curves were drawn, one for high and the other for low antibody concentration (10).

Oxidative metabolites were assessed by means of the quantification of urinary peroxide, thiobarbituric acid reactive substances (TBARS), and renal tissue thiols. Assessment of urinary peroxides was carried out by the FOX-2 method (ferrous ion xylenol orange assay), which oxidizes Fe^2+^ ions producing a purplish-blue complex (α = 4.3 x 10^4^ M^-1^ cm^-1^) (11,12). Urinary TBARS assessment enables the identification of the final products of the lipid peroxidation cascade that react with thiobarbituric acid in organic fluids (α = 1.56 x 10^5^ M^-1^ cm^-1^) (13). Renal tissue thiols were evaluated by means of the reaction with 2,2’-dinitro5-5’dithio- benzoic acid, DTNB (α = 13.6 x 10^3^ M^-1^ cm^-1^) (14,15).

Quantification of tubulointerstitial lesions was carried out using a scale raging from 0 to 4, as follows: 0 = normal tissue; 0.5 = small, focal areas of alteration; 1 = 5-25% of the cortex area affected; 2 = 25-50% of the cortex area affected; 3 = 50-75% of the cortex area affected; and 4 = more than 75% of the cortex area affected (16). Slides were observed in an Axioskop 40 optical microscope (Carl Zeiss, Jena, Germany).

### Statistical analyses

Results are presented as means ± standard deviation. Statistical analyses of the results were carried out using the analysis of variance (ANOVA), followed by Tukey test for multiple comparisons in Graph-Pad Prism version-3 for Windows^®^. Significance level was set at p < 0.05.

## RESULTS

### Physiological parameters

**Figure 1:**
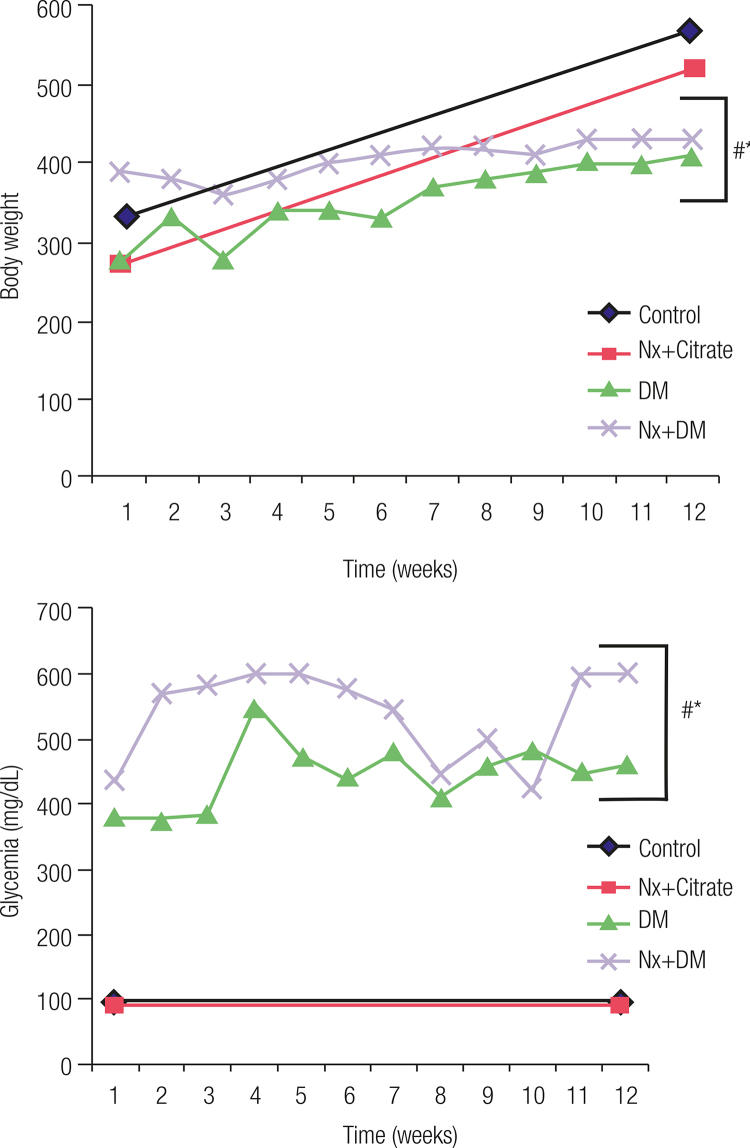
Body weight and blood sugar. # p < 0.05 vs. Control; * p < 0.05 vs. Nx+Citrate.

Table 1 shows that diabetic animals presented a significant increase in the intake of water (polydipsia) and feed (polyphagia) (p < 0.05). Nx showed significant increase in kidney weight when compared with animals that were not subjected to nephrectomy (p < 0.05); and the kidney/animal weight ration showed a significant difference between the nephrectomy group and the DM one (p < 0.05). When compared with the control group, diabetic animals presented significant differences, throughout the 12 weeks, in the variables weight (Figure 1A) and blood glucose (Figure 1B) (p < 0.05).

**Table 1:** General physiological parameters

Groups	Control (6)	Nx+Citrate (6)	DM (6)	Nx+DM (6)
Water intake/ 24h (mL)	23 ± 5	24 ± 6	116 ± 34^#^*	113 ± 35 ^#^*
Feed intake/ 24h (g)	14 ± 5	13 ± 5	26 ± 4^#^*	25 ± 8 ^#^*
Kidney weight (g)	1.4 ± 0.1	2.6 ± 0.5^#^	1.5 ± 0.1*	3.0 ± 0.4^#&^
Kidney weight/animal weight (x100)	0.28 ± 0.02	0.52 ± 0.13^#^	0.42 ± 0.05^#^	0.78 ± 0.07^#^*^&^

^#^ p < 0.05 vs. Control; * p < 0.05 vs. Nx+Citrate; ^&^ p < 0.05 vs. DM.

### Renal function

Table 2 shows increased urine flow in diabetic animals (polyuria). These animals also showed a reduction in urinary creatinine and significant increase in serum creatinine (p < 0.05), with reduced glomerular filtration rates evidenced by creatinine clearance (p < 0.05).

**Table 2:** Overall renal function

Groups	Control (6)	Nx+Citrate (6)	DM (6)	Nx+DM (6)
Urine flow (mL/min)	0.01 ± 0.02	0.01 ± 0.02	0.04 ± 0.03	0.06 ± 0.01
Urine creatinine (mg/dL)	52.8 ± 14.1	69.2 ± 7.6	20.4 ± 14.6^#^*	12.2 ± 2.7^#^*^&^
Serum creatinine (mg/dL)	0.22 ± 0.04	0.29 ± 0.04	0.55 ± 0.18^#^*	0.64 ± 0.05^#^*
Clcr/100 g (mL/min)	0.54 ± 0.14	0.51 ± 0.08	0.33 ± 0.09^#^*	0.32 ± 0.05^#^*

Clcr: creatinine clearance. ^#^ p < 0.05 vs. Control; * p < 0.05 vs. Nx+Citrate; ^&^ p < 0.05 vs. DM.

### Albuminuria

**Figure 2:**
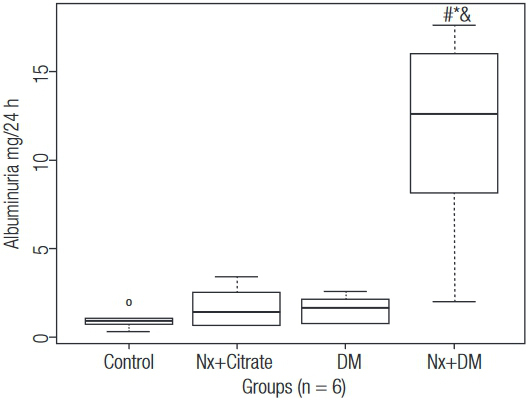
Albuminuria. # p < 0.05 vs. Control; * p < 0,05 vs. Nx+Citrate; & p < 0,05 vs DM.

Albuminuria (Figure 2) was significantly greater in diabetic animals that underwent nephrectomy (p < 0.05).

### Oxidative metabolites

**Figure 3:**
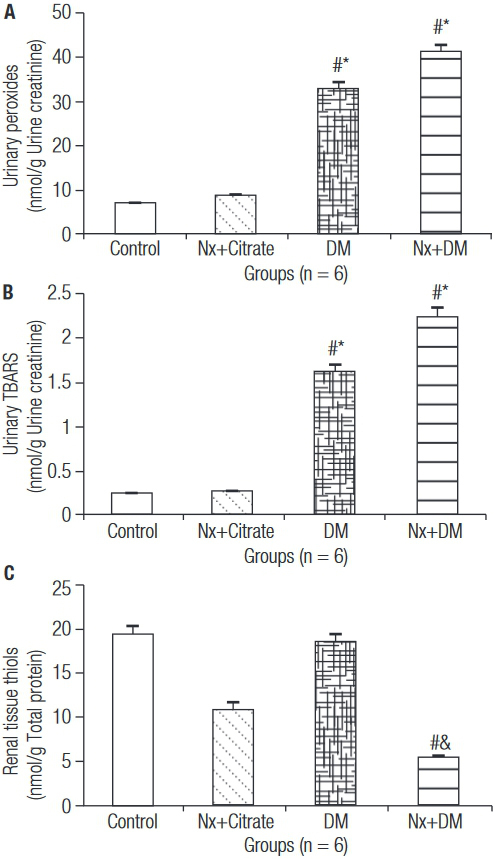
Oxidative profile. Urinary Peroxides (A), Urinary TBARS (B), and Renal Tissue Thiols (C). # p < 0.05 vs. Control; * p < 0.05 vs. Nx+Citrate; & p < 0.05 vs. DM.

The oxidative profile in the DM experimental model is shown in Figure 3. Increased urinary peroxides (Figure 3A) in the diabetic groups followed the increasing trend observed in TBARS (Figure 3B) (p < 0.05). The level of renal tissue thiols (Figure 3C) was inversely proportional, and showed the use of the antioxidant endogenous reserve in the group that showed the most aggressive lesion, the Nx+DM group (p < 0.05).

### Renal histology

**Figure 4:**
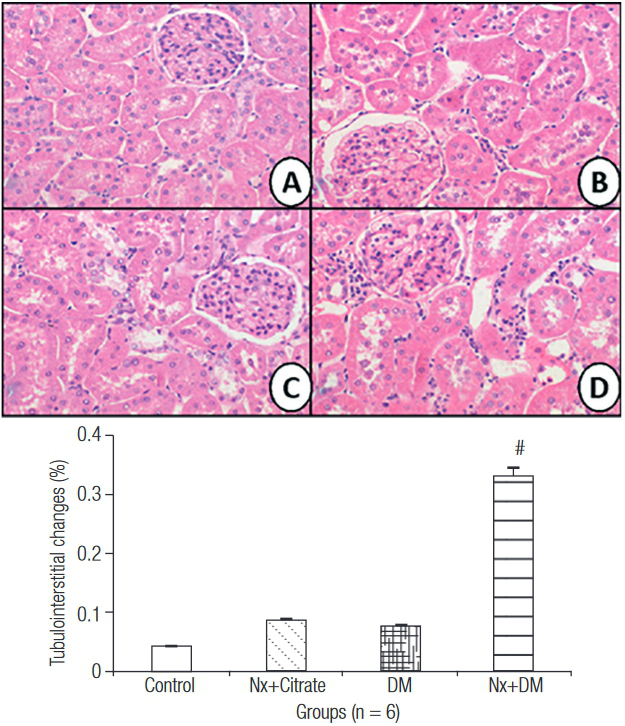
Histological sections and tubulointerstitial changes (400X magnification): Control (A); Nx+Citrate (B); DM (C) and Nx+DM (D). # p < 0.05 vs. Control.

Figure 4 shows the histological slides for the different groups. The Control group (A), Nx+Citrate (B), and DM (C) did not show histological changes. The Nx+DM (D) group showed discreet, segmental, and focal changes, such as tubulointerstitial fibrosis, evidenced by mesangial expansion, deposits of extracellular matrix, and tubulointerstitial damage (p < 0.05).

## DISCUSSION

Experimental induction of DM in Wistar rats by streptozotocin infusion led to classical signs of the disease, such as polydipsia, polyphagia, hyperglycemia, and weight loss. This finding demonstrates that maintenance of the hyperglycemic status favored the development of diabetic nephropathy evidenced by reduced creatinine clearance, albuminuria, and intense ROS release with reduction in the antioxidant reserve and focal changes in renal histology.

The first step in the study was the reproduction of the diabetes experimental model. The model was based on the administration of streptozotocin, a chemotherapeutic agent that degenerates pancreatic β cells, affecting insulin production. Chronic hyperglycemia, which is characteristic of this disease, ensued (17). It is known that chronic hyperglycemia favors energy imbalances with intense lipolysis, which was illustrated in this study by increased feed intake and progressive weight loss in the animals (17,18).

*In vivo* reproduction of early diabetic nephropathy by streptozotocin contributed with the hemodynamic changes induced by glomerular hyperfiltration and evidenced by physiological parameters observed, such as polydipsia, polyuria, and reduced GFR. The devastating effect of systemic hyperglycemia on glomerular filtration makes endothelial cells unable to modulate glucose transport through the plasma membrane, leading to excessive accumulation of intracellular glucose. The high levels of intracellular glucose stimulate the synthesis of cytokines, such as transforming growth factor β and vascular endothelial growth factor. These factors are involved in microvascular endothelial lesions, increasing glomerular permeability to macromolecules and add to the changes in glomerular hemodynamics mediated by vasodilating prostanoids and NO, which intensify vasodilation of the afferent arteriole in relation to the efferent one. Arteriolar imbalance leads to the final picture of glomerular hyperfiltration (3,7,19).

Changes related to glomerular dysfunction are represented by a persistent increase in hydrostatic intraglomerular pressure that forces the passage of fluids through the capillaries with loss of albumin into the ultrafiltrate (19,20). Glomerular dysfunction is characterized by synthesis and catabolism of several molecules, such as collagen IV and proteoglycans, which are responsible for the thickening of the basal glomerular membrane and changes in the negative charge of the podocytes, with subsequent loss of protein in the urine and glomerulosclerosis with reduced GFR (20,21). Additionally, animals that were subjected to nephrectomy confirmed the characteristics of renal lesions related to microvascular complications of diabetic nephropathy, which were evidenced by the increased kidney weight and relative weight (kidney weight/body weight). The persistent high glucose concentration in the glomerular region changes the structure of the basal membrane and promotes the expansion of the extracellular matrix in the mesangial area, which induces hyperplasia and hypertrophy of the tubular area (21).

In clinical practice, microalbuminuria is an indicator of glomerular filtration failure, which is identified 10 to 20 years after the onset of chronic hyperglycemia. Values of microalbuminuria that are considered predictive of the development of end-stage renal disease (ESRD) range from 30 to 300 mg/day (22). In this study, increased albuminuria in diabetic animals that underwent left nephrectomy intensified the progression of renal disease in the streptozotocin-induced DM model in rats. Diabetic nephropathy is defined by increased urinary excretion of albumin and reduced renal function (elevation in the concentration of plasma creatinine and reduced GFR), and in clinical practice, many times, it is necessary to determine renal replacement therapies, such as dialysis or kidney transplant (3,22). Diabetic nephropathy is the most common cause of ESRD in the USA, Japan, and Europe (1). In Brazil, the incidence of diabetic nephropathy reaches 31% in adult patients (23).

Chronic hyperglycemia is considered the most important factor in the generation of intracellular ROS. Diabetes complications are directly related with the inability of some cells to maintain intracellular glucose homeostasis, leading to the transport of large amounts of glucose to the inside of glomerular endothelium, mesangial and tubular epithelium cells, and intensifying glycolysis and excessive ROS production (5,6). The production of O2^∙-^ is the start of an oxidative lesion cascade mediated by H_2_O_2_, unbound iron, and generation of new free radicals, such as OH^∙-^ and peroxynitrite (ONOO^-^), which damage lipids, proteins, and nucleic acids (5,24).

The role of ROS in the experimental model of diabetic nephropathy was confirmed by the increased levels of urinary peroxides and TBARS in diabetic animals. H_2_O_2_ are highly capable of penetrating biological membranes and, once they are formed in excess, they induce the release of OH^.-^ and urinary TBARS, which are a direct quantification of lipid peroxidation catalyzed by unbound iron (6,24). The mitochondrial region is particularly susceptible to ROS-mediated lesions, showing dysfunctional ATP synthesis, unbalanced levels of intracellular calcium, and changes in mitochondrial permeability, which predispose the cell to necrosis and apoptosis (25).

As a response to excess ROS, several antioxidant systems are activated, including free radical scavengers and enzymatic systems. The enzymes glutathione peroxidase, catalase and other antioxidant enzymes catalyze the conversion of H_2_O_2_ into water (5,6). Hyperglycemia determines an imbalance in the consumption of antioxidant enzymes, which are predominantly represented by the enzymes superoxide dismutase, glutathione peroxidase, and catalase (5). Diabetic animals that underwent nephrectomy showed reduction in the levels of thiols, which indirectly represent the activity of the antioxidant enzyme. Some studies confirm the involvement of ROS in the lesion mechanism of diabetic nephropathy. An *in vivo* study with mesangial cells subjected to high concentrations of glucose demonstrated important generation of ROS (26). The administration of D-Saccharic acid 1,4 Lactone (DSL), an antioxidant found in citrus fruits and some vegetables, reduced ROS levels in rats with streptozotocin-induced diabetes (27).

The oxidative lesion caused by the increased generation of ROS and low level of antioxidant mediators has a fundamental role in the development of microvascular complications in renal diabetic disease. In this pathological scenario, renal tissue is damaged by excess intracellular glucose, which adds to the intense oxidation that induces cell lesion (2). Therefore, renal tissue of diabetic animals subjected to nephrectomy demonstrated slight, segmental and focal tubulointerstitial changes. In diabetic nephropathy, cells accumulate glycogen and collagen fibers in the mesangial region and in the basal membrane showing glomerulosclerosis, tubulointerstitial fibrosis, mesangial expansion, deposits of extracellular matrix, and tubulointerstitial changes (28,29). Therefore, this study reproduced the synergism found in the physiopathology of diabetic nephropathy by means of hemodynamic and oxidative mechanisms associated with histological changes.

In summary, the study enabled the understanding of the DM physiopathological scenario and microvascular complications of diabetic nephropathy by evaluating traditional renal function parameters and, mainly, by the perspective of oxidative stress. The study also confirmed that the occurrence of lipid peroxidation, probably associated with the intense consumption of antioxidant endogenous substrate in diabetic animals, may be main route for the lesions induced by high concentrations of intracellular glucose. The association between the DM model and the functional vulnerability status determined by uninephrectomy exactly reproduced the onset of classical diabetic nephropathy, with all its known clinical manifestations, and confirmed that this experimental model may employed in the investigation of possible lesions involved in this important 21^st^ century disease.
